# The role of high airway pressure and dynamic strain on ventilator-induced lung injury in a heterogeneous acute lung injury model

**DOI:** 10.1186/s40635-017-0138-1

**Published:** 2017-05-12

**Authors:** Sumeet V. Jain, Michaela Kollisch-Singule, Joshua Satalin, Quinn Searles, Luke Dombert, Osama Abdel-Razek, Natesh Yepuri, Antony Leonard, Angelika Gruessner, Penny Andrews, Fabeha Fazal, Qinghe Meng, Guirong Wang, Louis A. Gatto, Nader M. Habashi, Gary F. Nieman

**Affiliations:** 1 Department of Surgery, SUNY Upstate Medical University, 750 E Adams Street, Syracuse, NY 13210 USA; 2 Department of Pediatrics, University of Rochester Medical Center, Rochester, NY USA; 3 Department of Trauma Critical Care Medicine, R Adams Cowley Shock Trauma Center, University of Maryland School of Medicine, Baltimore, MD USA; 4 Department of Biological Sciences, SUNY Cortland, Cortland, NY USA

**Keywords:** Acute lung injury, Over-distension, Stress, Strain, Heterogeneous lung, Atelectasis, Acute respiratory distress syndrome (ARDS), Ventilator-induced lung injury (VILI), Heterogeneous lung inflation, Alveolar over-distension, Alveolar collapse and reexpansion, Static strain, Dynamic strain

## Abstract

**Background:**

Acute respiratory distress syndrome causes a heterogeneous lung injury with normal and acutely injured lung tissue in the same lung. Improperly adjusted mechanical ventilation can exacerbate ARDS causing a secondary ventilator-induced lung injury (VILI). We hypothesized that a peak airway pressure of 40 cmH_2_O (static strain) alone would not cause additional injury in either the normal or acutely injured lung tissue unless combined with high tidal volume (dynamic strain).

**Methods:**

Pigs were anesthetized, and heterogeneous acute lung injury (ALI) was created by Tween instillation via a bronchoscope to both diaphragmatic lung lobes. Tissue in all other lobes was normal. Airway pressure release ventilation was used to precisely regulate time and pressure at both inspiration and expiration. Animals were separated into two groups: (1) over-distension + high dynamic strain (OD + H_DS_, *n* = 6) and (2) over-distension + low dynamic strain (OD + L_DS_, *n* = 6). OD was caused by setting the inspiratory pressure at 40 cmH_2_O and dynamic strain was modified by changing the expiratory duration, which varied the tidal volume. Animals were ventilated for 6 h recording hemodynamics, lung function, and inflammatory mediators followed by an extensive necropsy.

**Results:**

In normal tissue (N_T_), OD + L_DS_ caused minimal histologic damage and a significant reduction in BALF total protein (*p* < 0.05) and MMP-9 activity (*p* < 0.05), as compared with OD + H_DS_. In acutely injured tissue (ALI_T_), OD + L_DS_ resulted in reduced histologic injury and pulmonary edema (*p* < 0.05), as compared with OD + H_DS_.

**Conclusions:**

Both N_T_ and ALI_T_ are resistant to VILI caused by OD alone, but when combined with a H_DS_, significant tissue injury develops.

## Background

Severe systemic inflammation and shock can result in the development of acute respiratory distress syndrome (ARDS), which remains a serious clinical problem associated with an unacceptably high mortality [1]. Currently, there are no effective treatments for ARDS, only supportive care via mechanical ventilation and adjuncts to therapy such as proning [2]. However, mechanical ventilation is a double-edged sword: although necessary for respiratory support, when set improperly, it can cause a secondary ventilator-induced lung injury (VILI) that can exacerbate ARDS mortality [3]. Thus, identifying the optimal ventilator settings necessary to minimize VILI has received a great deal of basic science [4] and clinical investigation [5].

Alveoli share walls with neighboring alveoli; this interdependent design equalizes airway pressure between adjacent alveoli, minimizing both collapse and over-distension (OD), as long as all alveoli are open and homogeneously ventilated [6]. However, this system is dependent on lung volume (decreased resistance in collateral channels with increase in lung volume) and largely defeated in conditions when lung volume is below functional residual capacity (FRC) [7].

Even in the normal lung, however, injurious mechanical ventilation over a period of time can cause surfactant deactivation and alveolar edema [8]. Surfactant deactivation and alveolar edema disrupt alveolar interdependence resulting in heterogeneous ventilation with open, collapsed, and edema-filled alveoli adjacent to each other. This structural alteration of alveolar microanatomy results in undue strain on alveolar walls. The applied force (i.e., tidal volume) in a heterogeneous lung causes an uneven stress with the lung tissue, known as stress concentrators, producing excessive alveolar strain, which is a primary mechanical mechanism of VILI [9–13]. Thus, if ventilator settings can be adjusted to convert heterogeneous to homogeneous ventilation, VILI will be reduced. However, this can be arduous, when in the heterogeneous lung there is both normal tissue (N_T_) and acutely injured lung tissue (ALI_T_), which must be ventilated with a single mechanical breath. In the heterogeneous lung, airway pressure must be sufficient to open alveoli with altered surfactant or edema, without causing over-distension of normal alveoli [14].

We have developed a novel heterogeneous lung injury model in which we control the exact location of N_T_ and ALI_T_. Using this model, we are able to measure the impact of the mechanical breath on both N_T_ and ALI_T_ within the same animal. We hypothesized that airway pressure sufficient to cause OD [40 cmH_2_O] would result in minimal damage to N_T_, provided there is a low dynamic strain (L_DS_). However, if this same high airway pressure were combined with a high dynamic strain (H_DS_) (i.e., large tidal volume), VILI would occur. We further hypothesized that high dynamic strain would exacerbate tissue damage in ALI_T_.

## Methods

All experiments were conducted with approval from the State University of New York Upstate Medical University Institutional Animal Care and Use Committee in accordance with ARRIVE guidelines.

### Surgical preparation and baseline measurements

Female Yorkshire pigs (30–35 kg) were anesthetized with an intravenous ketamine (90 mg/kg) and xylazine (10 mg/kg) solution. A 7.5-mm endotracheal tube (Harvard Apparatus) was placed through a tracheostomy and connected to a mechanical ventilator (Dräger Evita Infinity V500) with baseline settings set at a tidal volume (Vt) of 10 mL/kg, respiratory rate (RR) of 10 breaths/min, positive end-expiratory pressure (PEEP) of 5 cmH_2_O, and a fraction of inspired oxygen (FiO_2_) of 100%. A central venous catheter was placed in the external jugular and was used for measurement of central venous pressure (CVP) and fluid and medication administration. The right carotid artery was cannulated and used for arterial blood pressure monitoring and blood gas (ABG) measurements (cobas b 221, Roche). A PiCCO catheter (PULSION Medical Systems, Germany) placed in the right femoral artery was used for measurement of cardiac parameters and pulmonary edema. A Swan-Ganz catheter (Edwards Lifesciences, USA) was wedged into a pulmonary arteriole through the central venous catheter for pulmonary artery (Ppa) and pulmonary artery wedge (Ppw) pressure measurements. An esophageal balloon-tipped catheter (CooperSurgical, Trumbull, CT) was placed into the distal esophagus to measure esophageal pressure (Pes), which was used to calculate transpulmonary pressure (Ptp).

### Study protocol

**Fig. 1:**
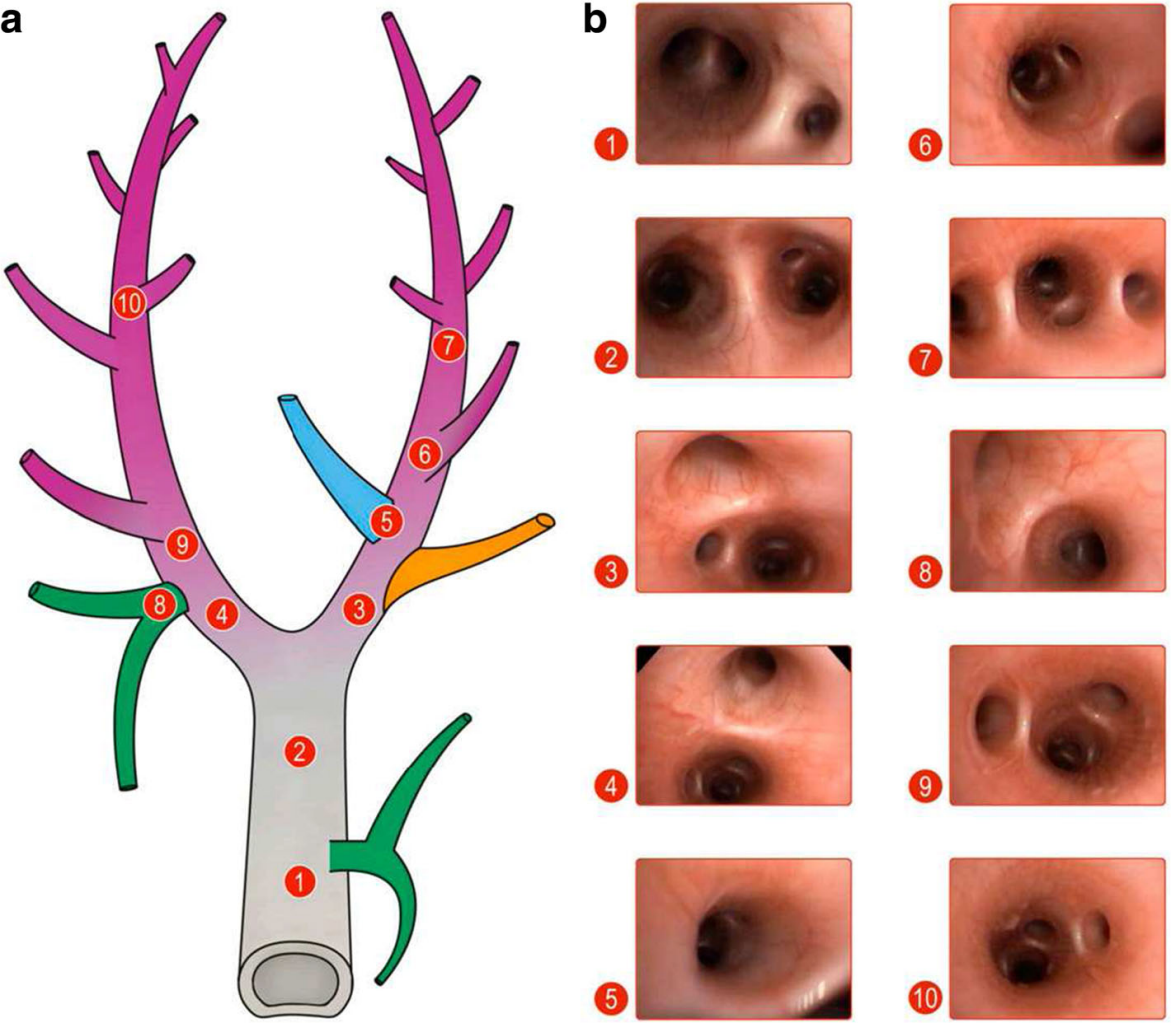
**a)** Schematic representing trachea as well as mainstem, lobar, and sublobar bronchi in pigs. Numbers label **b**) representative bronchoscopic images at those specific points in the tracheobronchial tree. The airway was visualized, and the bronchoscope was advanced down the right mainstem bronchus first, until reaching *point 6*. 0.75 mL/kg of 1% Tween was instilled at this point in order to specifically target dependent lung regions. The bronchoscope was withdrawn to the carina and then advanced down the left mainstem bronchus until reaching *point 9*, and another 0.75 mL/kg of 1% Tween was administered. The bronchoscope was completely withdrawn at this point and the animals randomized into two groups. (Reprinted with permission) [15]

**Fig. 2:**
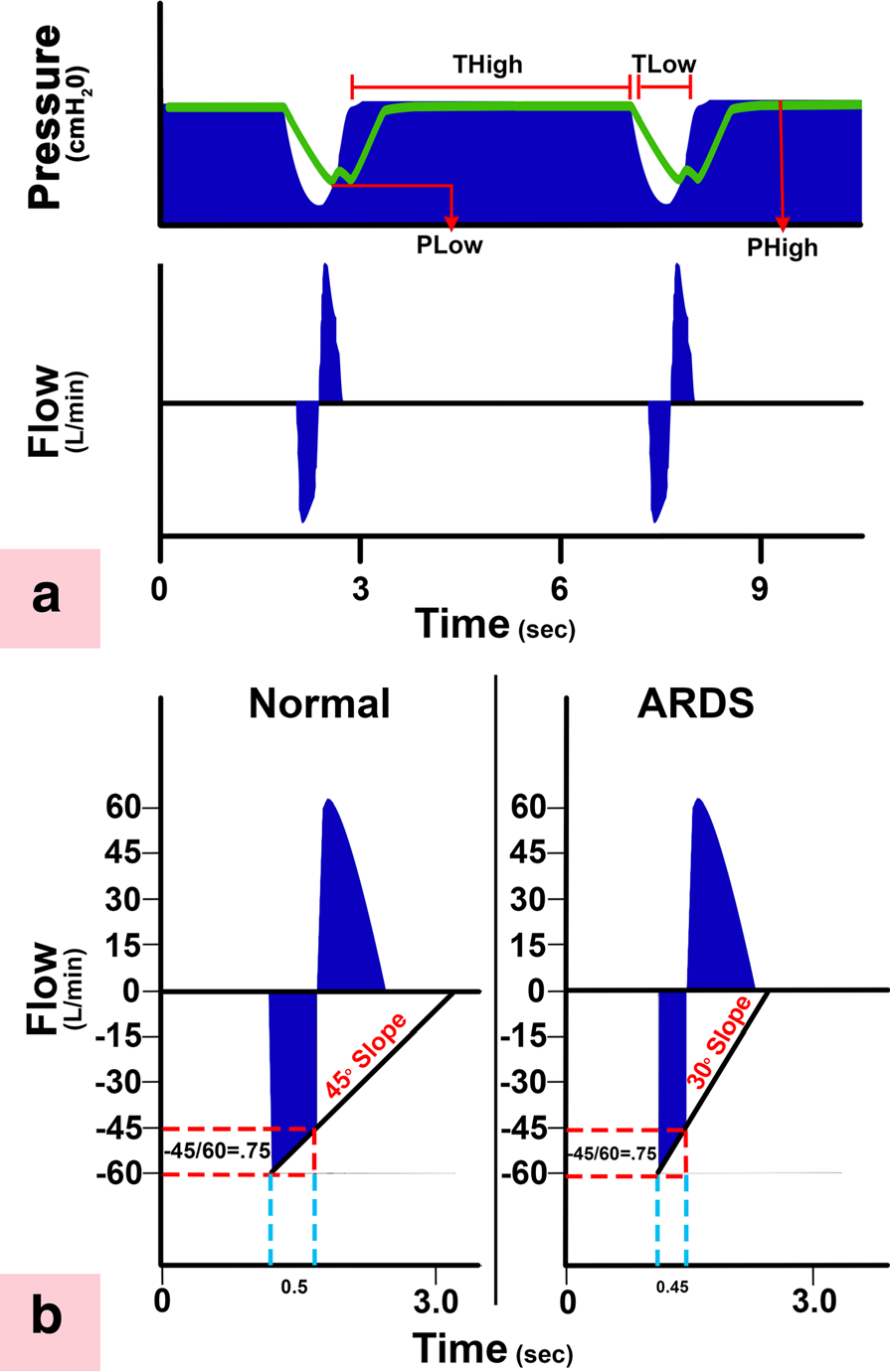
**a** Typical airway pressure release ventilation (APRV) airway *Pressure* and *Flow* curves. Correctly set APRV has a very brief duration at expiration (time at low pressure (*T*_*Low*_)) and extended inspiratory duration (time at high pressure (*T*_*High*_)) [17]. The T_High_ is ~90% of each breath. The two other APRV settings are the pressure at inspiration (*P*_*High*_) and at expiration (*P*_*Low*_). P_High_ is set sufficiently high to recruit and open alveoli, and P_Low_ is always set at 0 cmH_2_O to facilitate expiratory flow. However, T_Low_ is sufficiently short such that end-expiratory pressure (P_Low_) never reaches 0 cmH_2_O identified by the tracheal pressure (*green line*) maintaining a level of PEEP. **b** This figure summarizes our novel method to maintain alveolar stability by adaptively adjusting the expiratory duration as directed by the expiratory flow curve. The rate of lung collapse is seen in the *Normal* (slope 45°) and acutely injured lung (*ARDS*, slope 30°). ARDS causes a more rapid lung collapse due to decreased lung compliance. Our preliminary studies have shown that if the ratio of the peak expiratory flow (PEF, 60 L/min) to the end-expiratory flow (EEF, 45 L/min) (EEF/PEF) is equal to 75%, this expiratory duration (0.5 s) is sufficiently brief to stabilize alveoli [14, 38]. The lung with ARDS collapses more rapidly such that the EEF/PEF of 75% identifies a shorter expiratory duration of 0.45s is necessary to stabilize alveoli. Although the EEF/PEF is fixed, the expiratory duration is not, but rather is adaptive and will stabilize alveoli regardless of lung injury severity. Thus, this method of setting expiratory duration is adaptive to changes in lung pathophysiology and personalizes the mechanical breath to each individual patient. The values presented in this legend are just an example and may not reflect the actual values obtained in real life situations. (Reprinted with permission) [18]

Immediately prior to injury, the animals were switched to continuous positive airway pressure (CPAP) equivalent to their baseline plateau pressure (~19 cmH_2_O) for bronchoscopic Tween administration. Although Tween is not a clinical cause of ARDS, it rapidly deactivates pulmonary surfactant and thus simulates the well-known component of ARDS pathophysiology that of surfactant dysfunction. The airway was visualized with a bronchoscope, which was advanced down the right mainstem bronchus until reaching point 5 (E1, Fig. 1) [15]. A 1% Tween 20 detergent solution (0.75 mL/kg) was instilled to specifically target the dependent, diaphragmatic lung regions. The bronchoscope was withdrawn to the carina and then advanced down the left mainstem bronchus until reaching point 9 (E1, Fig. 1) [15], and an identical Tween dose was administered. The bronchoscope was then withdrawn and the animals randomized into two groups, OD + H_DS_ and OD + L_DS_. Airway pressure release ventilation (APRV) was used to deliver 40 cmH_2_O peak airway pressure (P_High_) during ~90% of each breath using an extended time at inspiration (T_High_) in both groups. This level of airway pressure has been shown to be associated with OD [16]. The dynamic strain levels (H_DS_ and L_DS_) were achieved by adjusting the duration of the release pressure (T_Low_). The end-expiratory pressure (P_Low_) was set at 0 to maximize expiratory flow and allow for maximum dynamic strain. An artistic depiction of the mechanical breath of APRV has been previously published (E2, Fig. 2) [17, 18].

The experimental variable in this study, dynamic strain, was adjusted by changes in T_Low_. To guide these changes, the expiratory flow curve was used as a tool to achieve specific targets, aimed at terminating the expiratory flow on each breath at two different points. In the OD + L_DS_ group, as the flow decelerated from its peak expiratory flow rate, T_Low_ was adjusted to terminate gas flow at 75% of that peak expiratory flow rate. For example, if a peak expiratory flow rate reached 100 L/min, the flow would be terminated once the deceleration of the flow rate reached 75 L/min. Another example of this method can be seen in Fig. 2b. The OD + H_DS_ group used the same method, but T_Low_ was adjusted to terminate gas flow at 25% of the peak expiratory flow rate. This ratio allowed three times as much expiratory flow to escape as compared to the OD + L_DS_, thus creating more end-expiratory collapse.

### Resuscitation

Lactated Ringer’s boluses were administered for mean arterial pressure (MAP) <65 mmHg after demonstration of fluid responsiveness with liver compression; if the animal was not fluid-responsive or required two boluses within 1 h, a norepinephrine infusion was started as an adjunct to keep MAP >65 mmHg. FiO_2_ was decreased if oxygenation improved with goal oxygen saturation >90%. No bicarbonate was given during the study.

### Physiologic and blood chemistry parameters

Cardiac output (CO), CVP, MAP, oxygen saturation, heart rate (HR), and temperature were continuously monitored (IntelliVue MP90, Philips Healthcare, Irvine, CA) and recorded hourly. Arterial blood gases, pH, and lactate were measured hourly with a Roche blood gas analyzer (cobas b 221, Basel, Switzerland). Ventilatory parameters and esophageal pressure measurements to calculate transpulmonary pressure measurements were also recorded hourly.

### Necropsy

After 6 h of ventilation, the protocol was terminated, the animals euthanized, and necropsy performed. Bronchoalveolar lavage fluid (BALF) and lung tissue were collected and frozen, lung tissue was fixed in formalin for histopathology, and edema was assessed by a lung tissue wet/dry weight ratio in both N_T_ and ALI_T_. The lungs were excised and inflated to 25 cmH_2_O, using stepwise increases in CPAP, for lung volume history standardization. A section of the apical lobe (non-Tween-injured tissue) and the right ventro-caudal lobe (Tween-injured tissue) were excised; one segment of each was submerged in formalin for histopathologic analysis, and another segment of each was snap frozen in liquid nitrogen. Normal saline (60 mL) was instilled separately into the right middle lobe (non-Tween-injured tissue) and the right dorso-caudal lobe (Tween-injured tissue) to collect BALF. The BALF was spun and the supernatant snap frozen.

### Inflammatory mediator measurement

Matrix metalloproteinase-9 (MMP-9) activity in BALF was determined using gelatin zymography, BALF total protein was determined using the bicinchoninic acid (BCA) method, and BALF surfactant protein A (SP-A) and B (SP-B) expression was determined using Western blot analysis.

Intercellular adhesion molecule (I-CAM-1), receptor of advanced glycation end products (RAGE), angiopoietin-2 (ANG-2), advanced glycosylation end product (AGER), type III procollagen (PCIII), E-cadherin, and NF-κB messenger RNA (mRNA) levels were all measured. Total RNA (2 μg) was isolated by the TRIzol method (Gibco BRL, Life Technologies) and converted to complementary DNA (cDNA) using an iScript cDNA Synthesis Kit (Bio-Rad Laboratories, Inc., Hercules, CA) following the manufacturer’s instructions. Then PCR was performed on a thermocycler (Thermo Electron Corp.). Amplification of cDNA was carried out in 20 μL solution containing 1 μL cDNA, 0.25 μM primer pairs for I-CAM-1, RAGE, ANG-2, AGER, PCIII, E-cadherin, NF-κB, and β-actin, and 10 μL of the DreamTaq Green PCR Master Mix (Thermo Fisher Scientific).

The PCR consisted of initial denaturation at 95 °C for 3 min, followed by 38 reaction cycles (30 s at 95 °C, 30 s at 55 °C, and 20 s at 72 °C) and a final cycle at 72 °C for 10 min. β-Actin was used as internal control. The amplified PCR products were separated by agarose gel electrophoresis and visualized with ethidium bromide. The abundance of each target mRNA was detected and normalized to that of β-actin mRNA.

Lung tissues were lysed in radioimmune precipitation (RIPA) buffer containing 50 mM Tris-HCl, pH 7.4; 150 mM NaCl; 0.25 mM EDTA, pH 8.0; 1% deoxycholic acid; 1% Triton X-100; 5 mM NaF; and 1 mM sodium orthovanadate supplemented with complete protease inhibitors (Sigma). Equal amounts of protein from the tissue lysates as determined by the BCA protein assay were subjected to SDS-PAGE and then transferred onto nitrocellulose membranes. The residual binding sites on the membrane were blocked by incubating with 5% (w/v) nonfat dry milk in TBST (10 mM Tris, pH 8.0; 150 mM NaCl; 0.05% Tween 20) for 1 h at room temperature. The membranes were subsequently incubated with the indicated antibodies and developed using an enhanced chemiluminescence (ECL) method, as described by Fazal et al. [19]. Polyclonal antibody to β-actin was from Santa Cruz Biotechnology, and rabbit polyclonal antibody for BiP/GRP78 was from Abcam. The nitrocellulose membranes were from Bio-Rad. ECL Plus Western Blotting Substrate and BCA Protein Assay Kit were from Pierce.

### Statistics

Depending on the underlying normal distribution, quantitative data are reported as mean with standard deviation. For the analyses of the developments over time, a general linear mixed model was used to compute differences within and between treatment groups. Pairwise comparisons were adjusted according to Tukey. For the comparison of two groups, a Student’s *t* test was used. A *p* < 0.05 was considered significant. Analysis was performed using SAS 9.4 (Cary, NC) and JMP 10 (Cary, NC).

## Results

There was no significant difference between groups in cardiac output (CO), mean arterial pressure (MAP), central venous pressure (CVP), arterial pH, serum lactate, or the volume of fluids and norepinephrine given (*p* > 0.05; Table 1). However, in both groups, there was a significant decrease over time in CO and MAP and increase in CVP, lactate, norepinephrine, and fluid volume (*p* < 0.05; Table 1).

**Table 1:** Hemodynamic and resuscitation data in animals with lung over-distension (OD) combined with either high (H_DS_) or low (L_DS_) dynamic strain. Cardiac output (CO, L/min), mean arterial blood pressure (MAP, mmHg), serum lactate concentration (lactate, mmol/L), norepinephrine (Norepi, mcg/min) and volume of crystalloid delivered (fluids, mL/kg). Data mean ± SD

		BL	T0	T1	T2	T3	T4	T5	T6
CO (L/min)	OD H_DS_	4.2 ± 1.4	3.5 ± 1.0	3.2 ± 1.1	3.4 ± 1.1	2.9 ± 0.6	2.8 ± 0.3	2.8 ± 0.5	2.7 ± 0.4
	OD L_DS_	4.9 ± 0.9	2.8 ± 0.8	2.6 ± 0.4	2.3 ± 0.3	2.4 ± 0.4	2.4 ± 0.6	2.7 ± 0.6	2.7 ± 0.6
MAP (mmHg)	OD H_DS_^†^	107.5 ± 13.6	83.5 ± 15.6	74.8 ± 5.7	78.3 ± 9.9	80.8 ± 12.2	80.5 ± 11.3	72.5 ± 7.6	60.7 ± 19.1
	OD L_DS_^†^	107.3 ± 21.7	83.0 ± 15.8	76.5 ± 7.2	67.3 ± 8.1	74.8 ± 25.7	72.3 ± 10.4	73.8 ± 11.0	70.3 ± 11.3
CVP (mmHg)	OD H_DS_^†^	6.5 ± 3.5	10.2 ± 2.1	10.8 ± 2.9	11.5 ± 2.1	11.2 ± 2.4	11.3 ± 2.2	11.0 ± 2.3	11.5 ± 2.9
	OD L_DS_^†^	6.2 ± 2.9	11.8 ± 3.1	13.5 ± 2.4	14.2 ± 2.1	13.0 ± 4.6	14.7 ± 4.7	16.8 ± 2.2	15.8 ± 3.8
Lactate (mmol/L)	OD H_DS_^†^	2.1 ± 0.6	4.5 ± 1.3	7.2 ± 1.2	8.9 ± 1.1	9.1 ± 1.9	9.4 ± 2.9	9.5 ± 3.1	9.5 ± 2.7
	OD L_DS_^†^	1.9 ± 0.8	3.7 ± 1.6	5.6 ± 1.5	8.5 ± 1.6	9.7 ± 2.9	10.7 ± 3.1	10.9 ± 3.5	11.4 ± 3.9
Norepi (mcg/min)	OD H_DS_^†^	0.0 ± 0.0	0.0 ± 0.0	1.3 ± 2.0	1.6 ± 2.5	4.1 ± 4.3	5.1 ± 5.3	4.8 ± 5.4	2.5 ± 5.5
	OD L_DS_^†^	0.0 ± 0.0	0.6 ± 1.6	0.6 ± 1.6	0.6 ± 1.6	3.5 ± 4.6	4.1 ± 5.2	4.1 ± 5.2	4.1 ± 5.2
Fluids (mL/h)	OD H_DS_^†^	1542 ± 772	2435 ± 890	3211 ± 1578	4749 ± 1932	5636 ± 2233	6191 ± 2338	7200 ± 2592	7949 ± 2653
	OD L_DS_^†^	1351 ± 635	2444 ± 1063	3459 ± 1310	5127 ± 1557	6766 ± 1880	8274 ± 1864	9644 ± 2004	10,901 ± 1993

^†^Significant difference within group over time

Inspiratory pressure was elevated to the same level (40 cmH_2_O) in both groups for the entire experiment (Table 2). There was a significant increase in end-expiratory pressure (EEP) (12.9[2.7] vs 3.0[1.2] cmH_2_O) and P/F ratio (333.0[156] vs 162.0[124]) in the OD + L_DS_ group as compared with the OD + H_DS_ group (mean[SD]; *p* < 0.05; Table 2). Driving pressure (35.6[2.4] vs 26.8[3.1] cmH_2_O), Vt/kg, and T_Low_ were significantly greater in the OD + H_DS_ group as compared with the OD + L_DS_ group (mean[SD]; *p* < 0.05; Table 2).

**Table 2:** Lung function data in animals with lung over-distension (OD) combined with either high (H_DS_) or low (H_DS_) dynamic strain

		BL	T0	T1	T2	T3	T4	T5	T6
Pplat (cmH_2_O)	OD H_DS_	18.2 ± 1.47	40 ± 0.0	40 ± 0.0	40 ± 0.0	40 ± 0.0	40 ± 0.0	40 ± 0.0	40 ± 0.0
	OD L_DS_	19.5 ± 1.64	40 ± 0.0	40 ± 0.0	40 ± 0.0	40 ± 0.0	40 ± 0.0	40 ± 0.0	40 ± 0.0
Pmean (cmH_2_O)	OD H_DS_	9.9 ± 1.0	29.7 ± 3.7^‡^	30.2 ± 2.6^‡^	31.0 ± 1.9^‡^	32.0 ± 3.0^‡^	32.7 ± 2.7^‡^	32.7 ± 2.3	32.7 ± 2.3^‡^
	OD L_DS_	11.0 ± 1.5	36.3 ± 0.5^‡^	36.0 ± 1.1^‡^	36.2 ± 1.3^‡^	36.0 ± 0.6^‡^	35.8 ± 1.0^‡^	35.8 ± 1.0^‡^	35.8 ± 1.0^‡^
EEP (cmH_2_O)	OD H_DS_	5 ± 0.0	2.5 ± 1.2^‡^	2.7 ± 1.1^‡^	2.7 ± 1.0^‡^	3.2 ± 1.6^‡^	3.3 ± 1.3^‡^	3.1 ± 1.0^‡^	3.0 ± 1.2^‡^
	OD L_DS_	5 ± 0.0	11.7 ± 2.8^‡^	12.2 ± 3.2^‡^	12.3 ± 4.0^‡^	12.7 ± 3.2^‡^	13.2 ± 3.1^‡^	12.7 ± 3.6^‡^	12.9 ± 2.7^‡^
Driving pressure (cmH_2_O)	OD H_DS_	14.4 ± 2.3	39.7 ± 6.2^‡^	37.3 ± 1.2^‡^	36.5 ± 1.9^‡^	36.3 ± 3.0^‡^	35.8 ± 2.7^‡^	36.1 ± 2.5^‡^	35.6 ± 2.4^‡^
	OD L_DS_	15.4 ± 2.8	28.3 ± 2.6^‡^	27.7 ± 2.6^‡^	27.6 ± 4.0^‡^	26.3 ± 2.1^‡^	26.4 ± 2.5^‡^	25.6 ± 2.9^‡^	26.8 ± 3.1^‡^
Vt (mL/breath)	OD H_DS_	424 ± 100	1067 ± 441	1112 ± 453	1038 ± 552	954 ± 580	920 ± 546	885 ± 500	878 ± 499
	OD L_DS_	460 ± 65	522 ± 153	658 ± 198	718 ± 230	722 ± 250	727 ± 260	726 ± 265	725 ± 156
Vt/kg (mL/kg/breath)	OD H_DS_^†^	10.0 ± 0.0	25.3 ± 3.9^‡^	26.8 ± 5.1^‡^	24.8 ± 9.9^‡^	22.8 ± 10.1^‡^	22.0 ± 9.3^‡^	21.1 ± 8.4^‡^	21.0 ± 8.5^‡^
	OD L_DS_^†^	10.1 ± 0.1	11.5 ± 3.4^‡^	14.3 ± 3.1^‡^	15.6 ± 3.8^‡^	15.7 ± 4.4^‡^	15.7 ± 4.4^‡^	15.7 ± 4.4^‡^	15.7 ± 4.2^‡^
T_Low_(s)	OD H_D_^†^	N/A	1.6 ± 0.3^‡^	1.7 ± 0.4^‡^	1.5 ± 0.6^‡^	1.4 ± 0.6^‡^	1.3 ± 0.6^‡^	1.3 ± 0.6^‡^	1.3 ± 0.6^‡^
	OD L_DS_^†^	N/A	0.5 ± 0.1^‡^	0.63 ± 0.2^‡^	0.64 ± 0.1^‡^	0.62 ± 0.1 ^‡^	0.62 ± 0.2^‡^	0.63 ± 0.2^‡^	0.63 ± 0.2^‡^
Resistance(cmH_2_O/L/s)	OD H_DS_	11.3 ± 3.0	19.1 ± 5.0	19.5 ± 5.0	21.5 ± 7.7	21.5 ± 6.9	21.4 ± 7.1	21.7 ± 6.2	21.9 ± 6.6
	OD L_DS_	12.4 ± 3.9	18.0 ± 5.5	17.8 ± 5.8	17.3 ± 5.8	17.4 ± 6.1	17.8 ± 6.5	17.9 ± 6.5	17.7 ± 6.4
Compliance (mL/cmH_2_O)	OD H_DS_	30.0 ± 7.2	26.3 ± 6.4	30.0 ± 12.2	28.8 ± 15.7	28.2 ± 18.1	27.8 ± 17.5	26.2 ± 15.6	24.5 ± 14.2
	OD L_DS_	30.6 ± 6.7	18.7 ± 6.3	24.3 ± 8.6	27.1 ± 10.6	24.6 ± 10.5	27.9 ± 10.3	29.2 ± 12.1	27.8 ± 11.0
Ptp (cmH_2_O/respiratory cycle)	OD H_DS_	12.5 ± 1.8	34.2 ± 2.5	34.2 ± 2.1	33.7 ± 2.9	32.3 ± 5.0	33.0 ± 3.3	32.7 ± 3.3	33.6 ± 3.7
	OD L_DS_	15.1 ± 5.0	30.9 ± 7.8	30.7 ± 7.6	30.7 ± 7.6	31.0 ± 7.8	30.8 ± 7.6	30.3 ± 7.9	30.2 ± 7.8
El (cmH_2_O/L)	OD H_DS_	21.6 ± 2.9	33.6 ± 10.7	33.0 ± 12.1	42.3 ± 33.1	40.3 ± 25.4	41.5 ± 22.4	41.9 ± 22.4	44.4 ± 24.9
	OD L_DS_	22.6 ± 8.5	49.8 ± 17.5	45.2 ± 20.2	43.6 ± 21.5	43.3 ± 22.0	40.9 ± 23.7	36.3 ± 27.6	39.3 ± 21.8
Ecw (cmH_2_O/L)	OD H_DS_	10.3 ± 4.5	6.6 ± 4.5	5.1 ± 3.2	7.3 ± 4.5	9.8 ± 7.7	9.5 ± 7.0	9.6 ± 6.6	8.5 ± 7.0
	OD L_DS_	10.6 ± 5.7	8.3 ± 5.7	7.6 ± 3.4	7.2 ± 3.6	7.0 ± 4.1	7.0 ± 4.9	7.7 ± 5.4	7.2 ± 4.5
pH	OD H_DS_	7.49 ± 0.1	7.54 ± 0.1	7.50 ± 0.1	7.48 ± 0.1	7.44 ± 0.1	7.42 ± 0.1	7.39 ± 0.1	7.38 ± 0.1
	OD L_DS_	7.44 ± 0.1	7.37 ± 0.1	7.41 ± 0.1	7.40 ± 0.1	7.38 ± 0.1	7.37 ± 0.1	7.36 ± 0.1	7.35 ± 0.1
PaO_2_ (mmHg)	OD H_DS_	528.5 ± 99	100.2 ± 77	96.1 ± 61	77.2 ± 52	86.9 ± 71	94.7 ± 65	70.1 ± 28	67.5 ± 24
	OD L_DS_	497.8 ± 93	146.2 ± 136	242.7 ± 154	113.2 ± 52	83.5 ± 22	87.3 ± 14	86.3 ± 19	90.6 ± 17
PaCO_2_ (mmHg)	OD H_DS_	37.5 ± 3.7	33.1 ± 6.3	25.7 ± 7.4	28.9 ± 17.3	30.6 ± 18.8	32.7 ± 19.2	33.8 ± 18.7	34.0 ± 20.9
	OD L_DS_	39.3 ± 5.6	41.9 ± 12.2	31.7 ± 10.7	29.2 ± 15.3	29.2 ± 14.0	28.5 ± 12.4	27.8 ± 14.9	28.9 ± 15.3
FiO_2_ (%)	OD H_DS_^†^	1.00 ± 0.0	1.00 ± 0.0	0.83 ± 0.20	0.72 ± 0.30	0.72 ± 0.30	0.70 ± 0.30	0.64 ± 0.30	0.64 ± 0.30
	OD L_DS_^†^	1.00 ± 0.0	1.00 ± 0.0	0.67 ± 0.20	0.37 ± 0.10	0.38 ± 0.30	0.43 ± 0.30	0.42 ± 0.30	0.37 ± 0.30
P/F (mmHg)	OD H_DS_^†^	528.5 ± 99	100.3 ± 77	143.6 ± 142^‡^	141.7 ± 115^‡^	155.6 ± 133^‡^	169.9 ± 126^‡^	166.5 ± 123^‡^	162.0 ± 124^‡^
	OD L_DS_^†^	459.2 ± 124	146.2 ± 136	367.2 ± 155^‡^	342.6 ± 167	322.5 ± 189	298.7 ± 158^‡^	311.4 ± 160^‡^	332.9 ± 156^‡^

^†^Significant difference within group over time, ^‡^significant difference between groups, ﻿N/A = Not applicable

Parameters are plateau airway pressure (Pplat, cmH_2_O), mean airway pressure (Pmean, cmH_2_O), end-expiratory pressure (EEP, cmH_2_O), driving pressure = tidal volume/compliance, tidal volume (Vt), the expiratory time during expiration (T_Low_, s), airway resistance (resistance, cmH_2_O/L/s), lung compliance (compliance, mL/cmH_2_O), transpulmonary pressure (Ptp, cmH_2_O/respiratory cycle), lung elastance (El, cmH_2_O/L), chest wall compliance (Ecw, cmH_2_O/L), arterial blood pH, PaO_2_, PaCO_2_, fraction of inspired oxygen (FiO_2_, %), and PaO_2_/FiO_2_ ratio (P/F). Data mean ± SEM

**Fig. 3:**
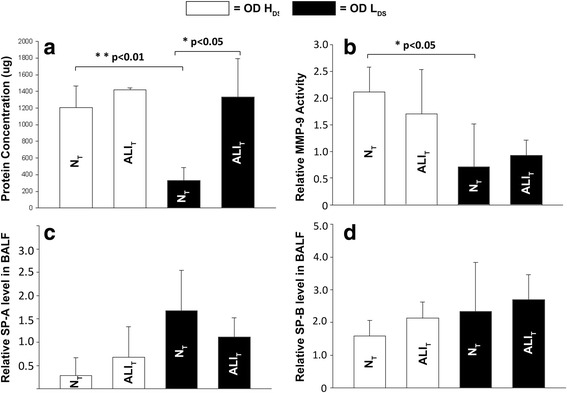
The effect of both high (*OD + H*_*DS*_) and low (*OD + L*_*DS*_) dynamic strain on total protein concentration, matrix metalloproteinase-9 (*MMP-9*) activity, and both surfactant protein A (*SP-A*) and B (*SP-B*) levels in bronchoalveolar lavage fluid (*BALF*) of normal (*N*_*T*_) and acutely injured (*ALI*_*T*_) lung tissue. **a** Low dynamic strain (OD + L_DS_) prevented the increase in total protein in normal lung tissue (N_T_). **b** Low dynamic strain (OD + L_DS_) prevented the increase in MMP-9 activity in N_T_. **c** High dynamic strain (OD + H_DS_) caused a decrease in SP-A in N_T_. **d** No differences in SP-B were seen with high or low dynamic strain or in normal or acutely injured lung tissue. Data mean ± SD

There was a decrease in the percent change of T_Low_ and Vt over time in the OD + H_DS_ group (Fig. 3). Since T_Low_ is adjusted based on lung mechanics, a reduction in T_Low_ may be required with a change in lung physiology and either adjusting T_Low_ or changes in lung compliance could cause a change in Vt. The percent change in both T_Low_ and Vt increased over time in the OD + L_DS_ group, suggesting improving lung pathology (Fig. 3).

**Fig. 4:**
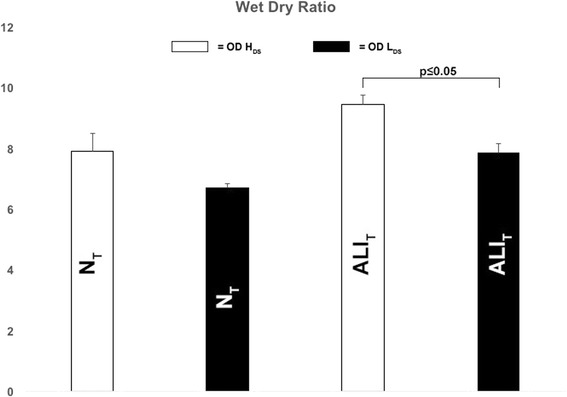
Pulmonary edema measured as a lung wet/dry weight ratio following ventilation with high (*OD + H*_*DS*_) and low (*OD + L*_*DS*_) dynamic strain and in normal (*N*_*T*_) and acutely injured (*ALI*_*T*_) lung tissue. Data mean ± SD

Figure 3 compares the impact of both OD + H_DS_ and OD + L_DS_ on both N_T_ and ALI_T_, caused by Tween instillation. BALF total protein concentration was elevated in ALI_T_ regardless of ventilation strategy; however, decreasing the dynamic strain (OD + L_DS_) significantly reduced the total protein concentration in N_T_ as compared to high dynamic strain (OD + H_DS_) ventilation (Fig. 3a). The OD + H_DS_ significantly increased MMP-9 activity in N_T_ as compared with OD + L_DS_ (Fig. 3b). There was no significant difference in surfactant protein A or B in either tissue type or with either ventilation strategy (Fig. 3c, d). OD + H_DS_ significantly increased pulmonary edema in ALI_T_ as compared with OD + L_DS_ (Fig. 4).

**Fig. 5:**
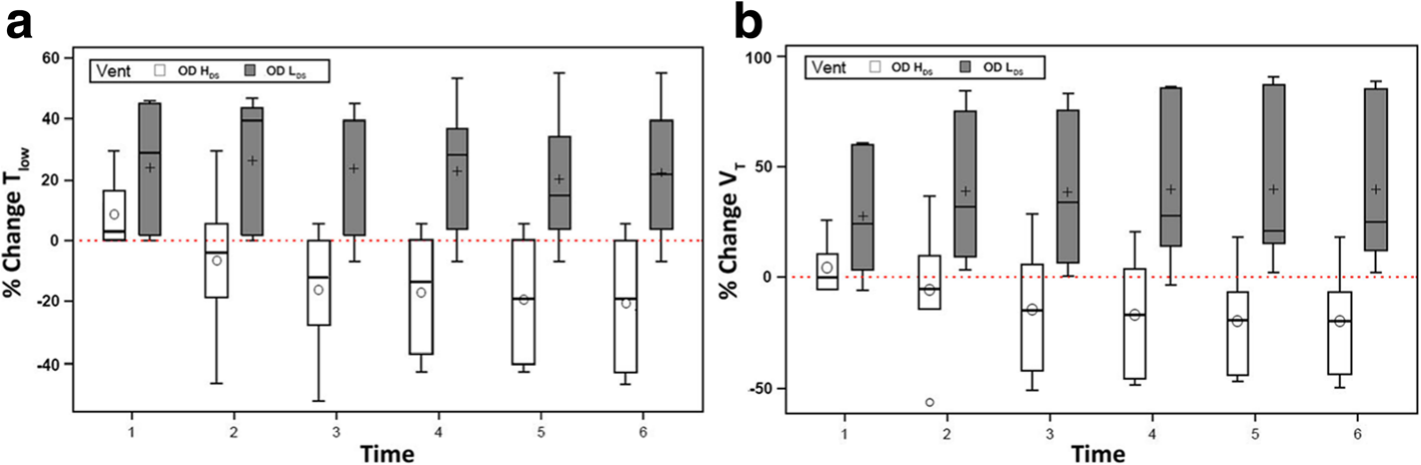
The percent change in both the duration at expiration (*T*_*Low*_), which is set by changes in lung physiology (see Fig. 2b) and the tidal volume (*Vt*) over time in both the high (*OD + H*_*DS*_) and low (*OD + L*_*DS*_) dynamic strain groups. **a** T_Low_ is set by changing lung physiology and will require shortening with increasing lung pathology/elastance. This suggests worsening lung injury with time in the OD + H_DS_ group but not in the OD + L_DS_ group. **b** Shortening T_Low_, with increasing lung pathology, will necessitate a reduction in Vt over time, which was seen in the OD + H_DS_ group but not in the OD + L_DS_ group. (○, mean of the distribution)

Multiple mediators of inflammation and injury (ICAM-1, NF-κB, RAGE, PCIII, and E-cadherin) were measured in lung tissue at necropsy, but none were significantly different either within or between groups. Protein levels of BiP/GRP78, an endoplasmic reticulum stress marker, were measured, and no significant difference was observed within or between groups (data not shown). The only significance was found in AGER, which was significantly greater in the N_T_: OD + L_DS_ group (1.67 ± 0.72 relative concentration) as compared with the N_T_: OD + H_DS_ group (0.64 ± 0.30 relative concentration, *p* < 0.05).

**Fig. 6:**
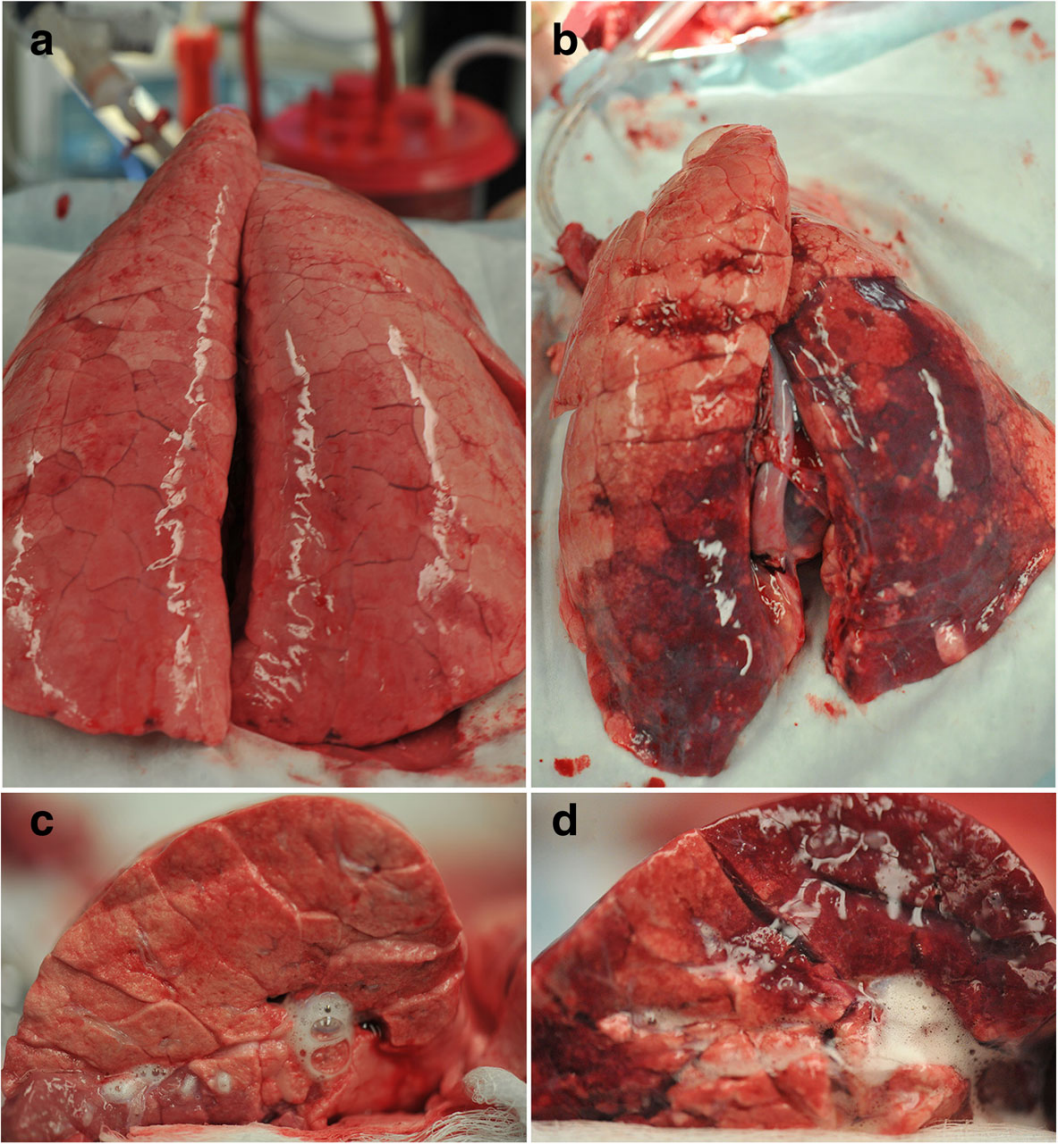
**a**–**d** (**6a**) low (OD +L_DS_)  and (**6b**) high (OD + H_DS_) dynamic strain and in normal (N_T_) and acutely injured (ALI_T_) lung tissue of the entire lung and the cut lung surface (**6c**–**d**) at necropsy. Lungs were inflated to 25 cmH_2_O to standardize lung volume history

**Fig. 7:**
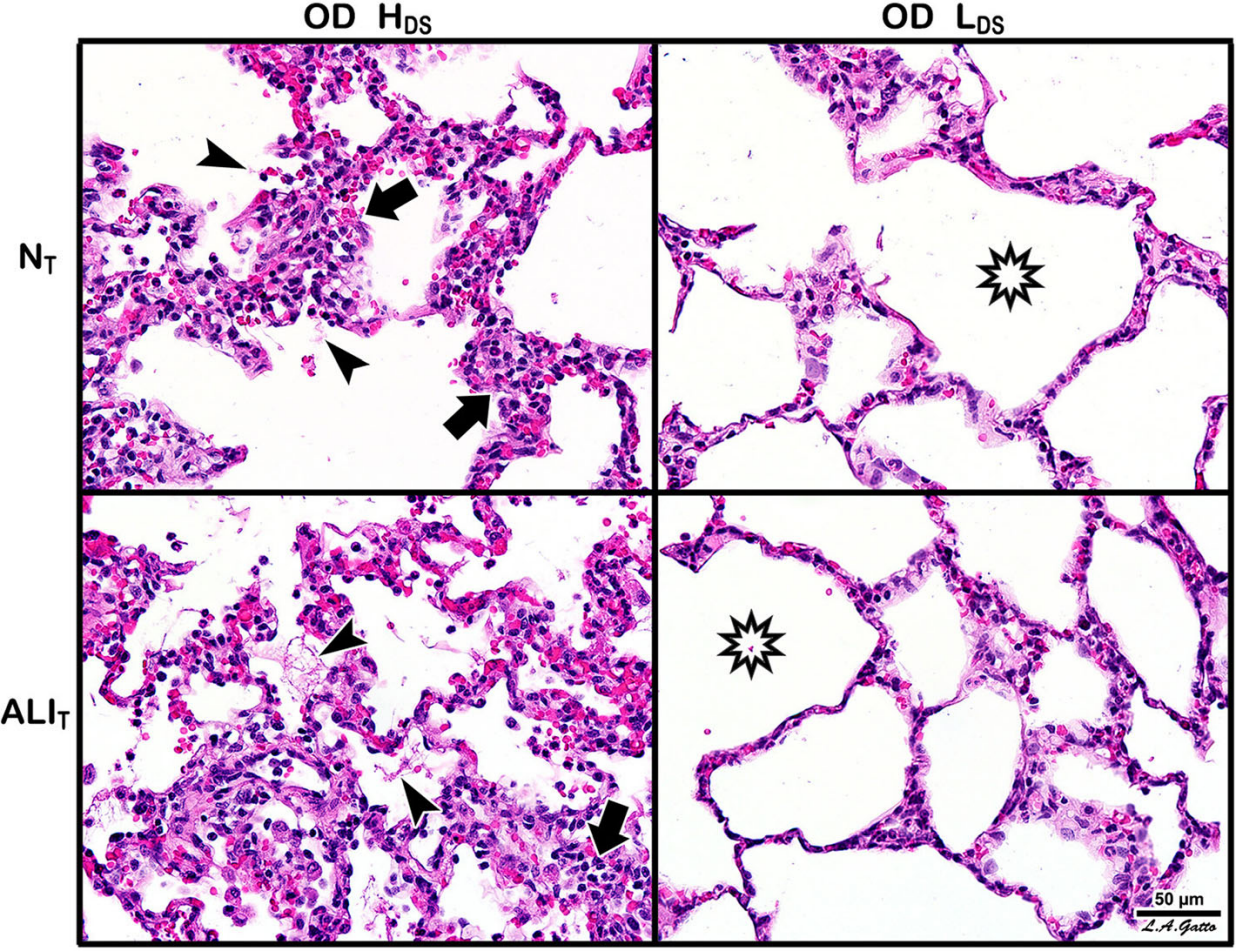
Histopathology following ventilation with high (*OD + H*_*DS*_) and low (*OD + L*_*DS*_) dynamic strain and in normal (*N*_*T*_) and acutely injured (*ALI*_*T*_) lung tissue. *Arrows* indicate cell infiltration, while *arrowheads* indicate the presence of fibrin deposits in the air compartment; both had higher incidence with H_DS_ irrespective of injury. *Stars* highlight alveolar patency, which was greater with L_DS_

Gross photographs at necropsy of the entire lung inflated to 25 cmH_2_O and the cut surface of the lobe in which Tween was instilled (i.e., diaphragmatic lobe) are seen in Fig. 5. The location of Tween instillation can be seen as dark red hepatized lung tissue on the diaphragmatic lung lobe and the cut lung surface in the high dynamic strain group (OD + H_DS_). The hepatized atelectatic lung tissue was not seen if the lung was ventilated at OD + L_DS_ for 6 h (Fig. 6). A similar protective effect of OD + L_DS_ as compared with OD + H_DS_ on both N_T_ and ALI_T_ was seen in histopathology (Fig. 7). This was shown by an increase in cellular infiltrates and fibrin deposition in the OD + H_DS_ group as compared to the OD + L_DS_ group.

## Discussion

The most important finding of this study is that ventilation of a heterogeneously injured lung with a peak inspiratory pressure of 40 cmH_2_O (an airway pressure that has been associated with lung over-distension) did not cause significant damage to normal lung tissue (i.e., the “baby lung”) [20] as long as dynamic strain was low. In addition, low dynamic strain in the presence of 40 cmH_2_O airway pressure showed significantly reduced pulmonary edema and histopathology, even in the tissue with acute lung injury. These data support our hypotheses that high static strain alone does not cause acute lung injury in normal tissue, nor does it exacerbate damage in lung tissue acutely injured by surfactant deactivation. This infers that the primary mechanism of VILI is alveolar collapse and reopening causing an excessive dynamic strain, and not high airway pressure causing tissue over-distension.

Heterogeneous lung injury is a hallmark of ARDS pathophysiology [10, 21], which can cause excessive alveolar strain leading to cellular damage in alveoli [6]. The lung with heterogeneous injury becomes exceedingly difficult to ventilate without causing a secondary VILI, since the airway pressure necessary to open acutely injured alveoli is far greater than that necessary to open normal alveoli. Our novel lung injury model allows us to study the impact of any mechanical breath on both the normal and acutely injured lung tissue. It has long been hypothesized that ventilating the ARDS lung with airway pressures greater than 30 cmH_2_O would cause damage (i.e., VILI) to the normal baby lung by over-distension [3]. However, normal homogeneously ventilated lung tissue should be protected from injury secondary to high inflation pressure due to alveolar interdependence as described by Jere Mead in 1970 [6]. Since alveoli share walls and are connected by pores of Kohn, there should be no pressure gradient across the walls of open, homogeneously inflated alveoli. With heterogeneous ventilation, alveoli lose stability with loss of interdependence. It was this theoretical basis and the work from Gattinoni’s group [8, 22–24] on which we based our hypothesis for this study.

Protti et al. [8] subjected normal pigs to mechanical ventilation for 54 h at the same global strain (i.e., near total lung capacity (TLC)) and titrated the strain by mainly dynamic and graduated to mainly static. The primary finding of this study was that high lung strain did not cause injury to normal lung tissue as long as it was not combined with a high dynamic strain. Our study confirms these findings using a clinically applicable heterogeneous lung injury model. The normal tissue in our animals suffered minimal damage, even with an airway pressure of 40 cmH_2_O, if dynamic strain was not excessive. In the normal tissue exposed to high as compared to low dynamic strain, there was significant increase in both alveolar total protein (suggesting increased capillary permeability) and MMP-9 activity (suggesting neutrophil activation). In addition, reducing the dynamic strain in the acutely injured lung tissue, even in the presence of 40 cmH_2_O airway pressure, reduced pulmonary edema and histologic injury.

What are the possible mechanisms for these observations? Biotrauma was not the likely difference in the histopathology, since only advanced glycosylation end product (AGER) was significantly different between groups, although increased MMP-9 activation might be part of the injury mechanism. It is possible that high dynamic strain is exacerbating surfactant dysfunction in both the normal and acutely injured lung tissue. Albert [25] has shown that surfactant loss, secondary to spontaneous or mechanical ventilation, can be the initiating event in the cascade of ALI progression that ultimately results in ARDS. If the surfactant film on the alveolar surface were compressed sufficiently, which would occur during expiration if lung volume fell far below normal functional residual capacity (FRC), the film would rupture, losing the ability of surfactant to lower alveolar surface tension [26, 27]. Webb and Tierney [28] hypothesized that the lung injury in animals without PEEP was caused by large Vt (i.e., high dynamic strain), deactivating or “wearing out” surfactant with excessive and repetitive compression of the surfactant.

Protti et al. investigated whether high global strain alone would cause lung edema or whether the type of strain (static vs dynamic) made a difference [8]. They found that high global strain per se did not necessarily cause pulmonary edema (i.e., low Vt and high PEEP), whereas edema and ARDS developed when the same global strain was delivered in a dynamic fashion (i.e., high Vt and low PEEP). They postulate that the mechanism of low dynamic strain-induced lung protection was by preventing disruption of the microvascular barrier. An alternate hypothesis is that the mechanism by which low dynamic strain prevents edema is preservation of surfactant function. It has been shown that deactivation of surfactant with a detergent aerosol resulted in a large change in alveolar volume with ventilation (i.e., high alveolar dynamic strain), which was associated with development of pulmonary edema [29, 30]. Since there was no increase in vascular permeability, the mechanism of edema formation was believed to be due to an increase in alveolar surface tension [30]. High alveolar surface tension decreases interstitial hydrostatic pressure increasing the pressure gradient between the capillary lumen and the interstitium, greatly accelerating fluid flux out of the vasculature into the alveolar lumen [31]. Although we did not show a difference in surfactant protein A or B concentration with low dynamic strain, it is possible that the ability for surfactant to lower surface tension was nevertheless impaired.

This paper improves our mechanistic understanding of lung protection using a ventilation strategy combining APRV, when used as an open-valve CPAP with a brief pressure release. If the pressure release phase is sufficiently brief, alveoli will be stabilized by two mechanisms: *pressure* and *time*. If the brief pressure release is set to less than the fastest alveolar collapse time constant, the alveolus does not have enough time to derecruit, and a *time-controlled alveolar stability* is generated. In addition, since the lung does not have time to fully collapse, an end-expiratory pressure is maintained generating a *time-controlled PEEP*. Some authors have suggested that auto-PEEP, or in the case of this study a time-controlled PEEP, is inferior to set PEEP because of heterogeneous alveolar collapse time constants causing the slow time constant alveoli to over-distend and the fast time constant alveoli to collapse. However, this hypothesis is based on theoretical, nonbiologic studies, using a single compartment model with a test lung [32, 33].

In a ten-patient study comparing auto-PEEP to set PEEP, there was no difference in lung compliance with only slightly worse gas exchange with auto-PEEP [34]. These authors speculate that auto-PEEP is preferentially applied to the normal, compliant units with slow time constants and that the noncompliant, fast time constant units empty first, decreasing end-expiratory volume resulting in atelectasis and atelectrauma in the injured lung regions [32]. However, all of these studies rely on inverse I:E ratio where the timing expiratory duration cannot be directly or precisely controlled, resulting in an expiratory duration greater than 1 s. Although the expiratory duration with inverse I:E may be sufficient to generate some auto-PEEP, it would be too long to prevent fast time constant alveoli from emptying, which is typically <0.5 s in an adult patient [35–37].

Thus, inverse I:E would only stabilize alveoli with pressure in the form of auto-PEEP or a combination of auto- and set PEEP rather than a combination of time and pressure. In the present study, the baby lung and the Tween-injured lung represent maximum heterogeneity of alveolar collapse time constants. Both normal and acutely injured lung tissue (fast and slow collapse time constants) were exposed to 40 cmH_2_O airway pressure. If the expiratory duration was sufficiently brief, <0.5 s in the low dynamic strain group, both normal and acutely injured lung tissue were protected as compared with an expiratory duration that was extended to >1.0 s in the high dynamic strain group.

Furthermore, several animal mechanistic and efficacy models have compared time-controlled PEEP to set PEEP. Time-controlled PEEP precisely controls the expiratory duration (100th of second) using changes in lung physiology (i.e., the slope of the expiratory flow curve) (E2, Fig. 2), and studies suggest that the expiratory durations determined by this method are less than the fastest time constant, which are typically <0.5 s [37]. Studies from our laboratory using time-controlled PEEP (E2, Fig. 2) vs a set PEEP in clinically relevant animal models of ARDS resulted in superior gas exchange, compliance, surfactant preservation, less microstrain, and reduced lung inflammatory and histopathology injury. In particular, the histology showed significantly less atelectasis in the time-controlled PEEP vs set PEEP as well as less edema and intra-alveolar debris and alveolar septal thickening [13, 14, 38–41].

This study demonstrates how very small changes in the expiratory duration can have a very large impact on lung mechanics. Using the slope of the expiratory flow curve to set the expiratory duration at a peak expiratory flow/end-expiratory flow (PEF/EEF) ratio of either 25 or 75% (Fig. 2) caused an increase in expiratory duration from 1.3 ± 0.6 (25%) to 0.63 ± 0.2 (75%) s, a difference of only 0.67 s (Table 2, T_Low_, T6). A difference of only 0.67 s expiratory time raised the end-expiratory pressure (EEP) from 3.0 ± 1.2 (25%) to 12.9 ± 2.7 (75%) cmH_2_O (Table 2, EEP, T6). We postulate that this time-controlled PEEP, combined with an expiratory duration less than the collapse time constant of the alveolus [35], works additively or synergistically to stabilize the lung and prevent alveolar collapse and reopening [13, 14, 38]. Better lung inflation was suggested in the group with the PEF/EEF ratio set at 75% as an increase in the P/F ratio and PO_2_ at a lower FiO_2_, fall in lung elastance over time (Table 2), and improved gross lung (Fig. 6) and histologic (Fig. 7) appearance, as compared to the PEF/EEF ratio set at 25% (Table 2). All of this lung protection, plus improvement in BALF total protein and MMP-9 activity, occurred with a mean change in expiratory time of only 0.67 s.

## Conclusions

To our knowledge, this is the first study to test the impact of dynamic strain on tissue injury in both normal and acutely injured lung tissue in the same animal. The normal tissue was not seriously injured when ventilated for 6 h at an inspiratory pressure of 40 cmH_2_O as long as dynamic strain remained low. However, 40 cmH_2_O pressure when combined with high dynamic strain caused significant damage to normal tissue and exacerbated damage to the injured tissue. This study suggests that VILI will be reduced if alveoli are recruited and stabilized, even in the presence of high plateau pressures. The mechanism of protection is complex and may be a combination of surfactant function preservation and/or preserving vascular integrity reducing the increase in vascular permeability and alveolar flooding.

## Abbreviations

ALI: Acute lung injury
ALIT: Acutely injured tissue
ANG-2: Angiopoietin-2
APRV: Airway pressure release ventilation
ARDS: Acute respiratory distress syndrome
BALF: Bronchoalveolar lavage fluid
BCA: Bicinchoninic acid
CO: Cardiac output
CPAP: Continuous positive airway pressure
CVP: Central venous pressure
EEP: End-expiratory pressure
FRC: Functional residual capacity
HR: Heart rate
I-CAM-1: Intercellular adhesion molecule
MAP: Mean arterial pressure
MMP-9: Matrix metalloproteinase-9
N_T_: Normal tissue
OD + H_DS_: Over-distension + high dynamic strain
OD + L_DS_: Over-distension + low dynamic strain
P/F: PaO_2_/FiO_2_
PCIII: Type III procollagen
PEF/EEF: Peak expiratory flow/end-expiratory flow
Pes: Esophageal pressure
P_High_: High pressure
P_Low_: Low pressure
Ppa: Pulmonary artery pressure
Ppw: Pulmonary artery wedge pressure
Ptp: Transpulmonary pressure
RAGE: Receptor of advanced glycation end products
RR: Respiratory rate
SP: Surfactant protein
T_High_: Time at high pressure
T_Low_: Time at low pressure
VILI: Ventilator-induced lung injury
Vt: Tidal volume
